# ﻿Revision of the genus *Urvaschia* Hopp (Hemiptera, Lygaeoidea, Oxycarenidae), with descriptions of two new species from China and Nepal

**DOI:** 10.3897/zookeys.1123.87863

**Published:** 2022-10-04

**Authors:** Cuiqing Gao, Shiya Xiao, Előd Kondorosy

**Affiliations:** 1 Co-Innovation Center for Sustainable Forestry in Southern China, College of Forestry, Nanjing Forestry University, Long Pan Road, Nanjing, Jiangsu 210037, China Nanjing Forestry University Nanjing China; 2 Department of Conservation Biology, Georgikon Campus, Hungarian University of Agriculture and Life Sciences, H-8360 Keszthely, Hungary Hungarian University of Agriculture and Life Sciences Keszthely Hungary

**Keywords:** Asia, distribution, Heteroptera, key, *Microplax*, new combination, taxonomy, true bugs

## Abstract

The species of *Urvaschia* Hopp, 1987 are reviewed. The following taxonomic change is proposed: *Urvaschiaobscuripennis* (Kiritshenko, 1914), **comb. nov.** (transferred from *Microplax* Fieber, 1860). The genus *Urvaschia* Hopp is newly recorded from Afghanistan, China, Iran, and Tadzhikistan. Two new species of *Urvaschia*, *Urvaschiaconvexa***sp. nov.** and *U.recta***sp. nov.** are described from China and Nepal. A diagnosis of the genus, a key to all of the included species, habitus photographs, and male genitalia illustrations of selected species are presented.

## ﻿Introduction

The lygaeoid family Oxycarenidae (Hemiptera: Heteroptera) includes, until now, 27 genera and approximately 140 species worldwide (Dellapé and Henry 2022). The genus *Urvaschia* Hopp, 1987 (Hemiptera: Heteroptera: Lygaeoidea: Oxycarenidae) currently contains only one described species occurring in the high mountains of Nepal and Kashmir (Hopp 1987). The authors studied the Oxycarenidae material of several Eurasian collections and found two new species which are described. Furthermore, a species currently belonging to *Microplax* Fieber, 1860, is more closely related to *Urvaschiapterosticta* Hopp, 1987 than to any other known species.

## ﻿Materials and methods

Composite images were obtained with an M205FA Leica stereomicroscope and camera using the Leica Application Suite software (ver. 4.5.0). Localities were mapped using SimpleMappr (Shorthouse 2010).

Label data are cited verbatim, lines on the same label are divided by a slash (/), and different labels are divided by double slashes (//). Printed [pr] and handwritten [hw] texts are indicated. Details of male dissection methods and terminologies used in this article follow those given in Ashlock (1957) and O’Donnell (1991). The vein terminologies used in this article are those provided in Wootton and Betts (1986). All measurements in the text are given in millimetres.

### ﻿Abbreviations

**BMNH**Natural History Museum, London, United Kingdom;

**CEHI in TLMF** Collection Ernst Heiss in Tiroler Landesmuseum Ferdinandeum, Innsbruck, Austria;

**HNHM**Hungarian Natural History Museum, Budapest, Hungary;

**IZAS** Institute of Zoology, Academia Sinica, Beijing, China;

**NHMB**Naturhistorisches Museum, Basel, Switzerland;

**NKUM**Institute of Entomology, Nankai University, Tianjin, China;

**NMPC**National Museum of Natural History, Prague, Czech Republic;

**TNHM**Tianjin Natural History Museum, Tianjin, China;

**ZIN**Zoological Institute, Russian Academy of Sciences, St. Petersburg, Russia.

## ﻿Taxonomy

### ﻿ Urvaschia

Taxon classificationAnimaliaHemipteraOxycarenidae

Hopp, 1987

68A53176-BBD9-5D2E-B841-9B0D6D27AAF8

Figs 1 , 2 , 3 , 4

Urvaschia Hopp, 1987: 225–240; Slater and O’Donnell 1995: 78.

#### Type species.

*Urvaschiapterosticta* Hopp, 1987.

#### Diagnosis

**Figure 1.**
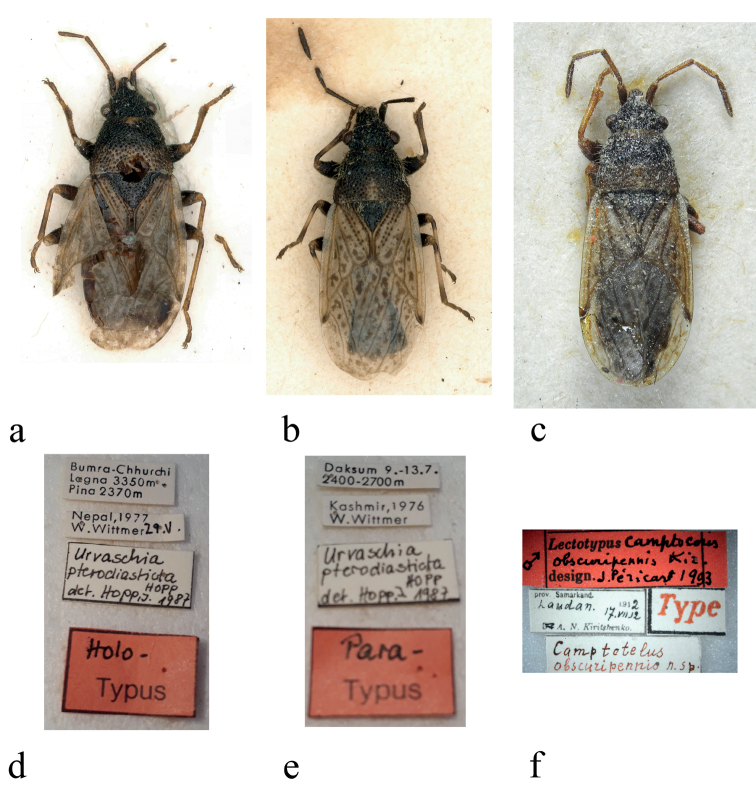
Type photographs **a, d***Urvaschiapterosticta*, holotype, habitus, and labels **b, e***Urvaschiapterosticta* paratype, habitus, and labels **c, f***Urvaschiaobscuripennis* comb. nov., habitus and type labels (photographed by F. Konstantinov, ZIN (**c, f**) and I. Zürcher, NHMB (**a, b, d, e**)).

**Figure 2.**
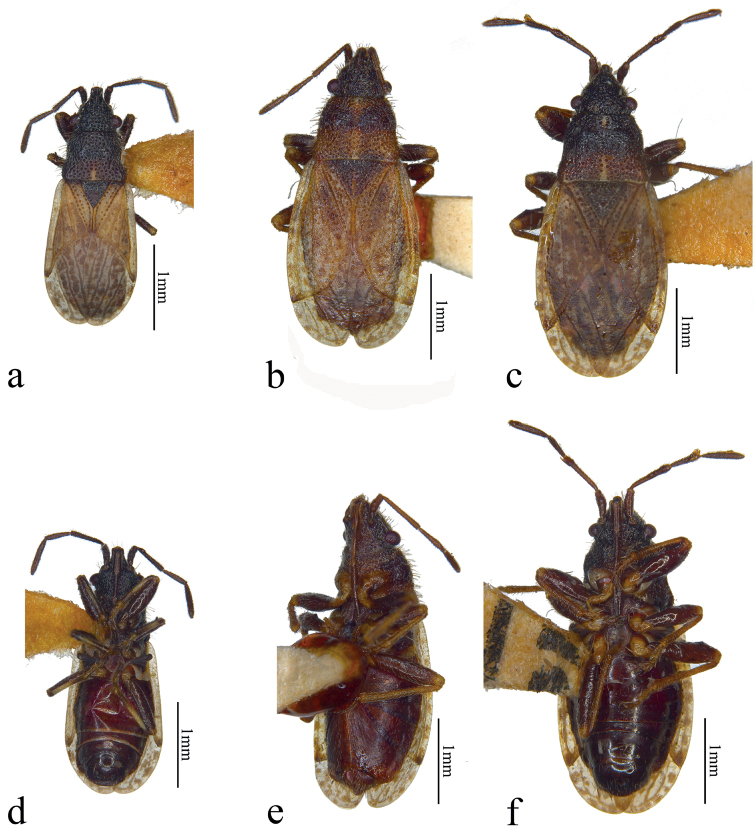
Dorsal and ventral views **a, d***Urvaschiaobscuripennis* comb. nov. **b, e***Urvaschiaconvexa* sp. nov., holotype **c, f***Urvaschiarecta* sp. nov., holotype.

**(modified from Hopp 1987) (Figs 1, 2).** Body elongate oval. Less than half length of first segment of antenna exceeding clypeus. Head short with eyes near to pronotum (less than one half diameter of eyes); bucculae short, only reaching base of antennae; labium almost reaching mesocoxae. Forewing slightly exceeding tip of abdomen; corium clearly punctate, at least between Cu vein and clavus; clavus punctate; membrane with thick veins, with distal ends fused to form four closed cells on membrane; corium and membrane between the veins covered with conspicuous brown spots. Profemur unarmed or sometimes with one very tiny spine.

#### Differential diagnosis.

*Urvaschia* differs from *Microplax* Fieber, 1860 by lacking any spine or with a very tiny spine at the distal part of the profemur (vs. one distinct spine and some tiny spines present at the distal part of profemur in *Microplax*); head short with a short postocular part which is less than 1/2 longitudinal diameter of the eyes (vs. head elongate with a long postocular part which is approximately as long as the diameter of eyes in *Microplax*); corium is clearly punctate and with many tiny spots (vs. corium lacking any punctures and unicolourous or with large spots in *Microplax*).

*Urvaschia* is also similar to *Camptotelus* Fieber, 1860 but it can be distinguished from the latter by bucculae not enlarged laterad, first segment of antennae exceeding clypeus, and clavus punctate (vs. the bucculae enlarged laterad, first segment of antennae not exceeding clypeus, and clavus impunctate in *Camptotelus*).

*Urvaschia* can be distinguished from *Leptodemus* Reuter, 1900 by the first segment of the antennae exceeding clypeus and the hemelytra punctate (vs. the first segment of antennae not exceeding clypeus and the hemelytra are impunctate in *Leptodemus*).

The key of Péricart (1999) contains all Palaearctic Oxycarenidae genera except *Urvaschia*. *Urvaschia* runs to couplet 16 (15) (to *Leptodemus*) but they differ in the above-mentioned features. The other possibility if we choose that the specimen has at least one tiny spine on the profemur, we run either to *Leptodemus* at couplet 22 (23) (again) or to *Microplax* at couplet 24 (25) if we choose “profemur has at least one distinct tooth”. Therefore, no described genus has identical characters shared with *Urvaschia*.

### ﻿ Urvaschia pterosticta

Taxon classificationAnimaliaHemipteraOxycarenidae

Hopp, 1987

253BAB1B-B1B1-5A6C-936C-97020796A5F8

Figs 1a, d , 4

Urvaschia pterosticta : Hopp, 1987: 226: original description; Slater and O’Donnell 1995: 78: catalogue.

#### Type material examined.

***Holotype*** (Fig. 1a, d) • Nepal ♂; Bumra-Chhurchi [pr] / Logna 3350m [pr] / Pina 2370m // Nepal, 1977 / W. Wittmer [pr] 29. V. [hw] // Urvaschia [hw] / pterodiasticta [hw] / HOPP [hw] / det. Hopp. I. 1987 [hw] // Holo- [hw] / Typus [pr] [red label] (NHMB).

#### Redescription.

***Colouration*.** Head black. Antennae blackish brown with segments II and III yellowish brown. Anterior lobe of pronotum black, with a yellow mid spot in the anterior margin; posterior lobe brown with darker punctures. Scutellum black. Corium pale yellowish brown, with sparse obscure pale brown spots including exocorium; veins thick and brown; apical angle of corium with single small blackish brown spot. Colour of membrane similar to corium, with dark brown spots on distal margin and between brown veins. Femora blackish brown; tibiae yellow with both ends brown; colour of tarsi similar to apices of tibiae.

***Structure*.** Head slightly declined, both dorsally and ventrally with very dense, deep, large punctures. Dorsal surface flat. Eyes slightly protruding laterally. Distance between posterior margin of eyes and anterior margin of pronotum approximately one fourth of diameter of eyes. Antennae covered with short dense oblique setae; apical 1/2 of segment I surpassing clypeus.

Pronotum trapezoid, swollen, calli slightly emergent. Anterior and posterior margin straight; lateral margins slightly arched. Pronotum covered with large, dense punctures. Clavus with three distinct shallow rows of large punctures, with middle row incomplete. Corium with scattered punctures between vein Cu and clavus; apical margin strongly concave, costal margin convex; apical angle elongated and narrow; total length of corium ~ 2/3 of hemelytra. Membrane relatively long and broad (Fig. 1a); membranal veins thick and obvious; apex of membrane surpassing abdomen. Fore femora slightly thickened, without any spines (Fig. 1a). Abdominal connexivum not exposed.

Pygophore (based on Hopp 1987): posterior margin of pygophore and cup-like sclerite fused; distal margin of cup-like sclerite without a deep incision. Parameres: outer projection large and rounded; inner projection very small and pointed.

#### Distribution.

Nepal (Hopp 1987) (Fig. 4).

#### Remarks.

The female paratype from Kashmir (Fig. 1b) of *U.pterosticta* has a straight and unicolourous exocorium, and the anterior margin of its pronotum and the antenna are uniformly dark. Therefore, it is identical with *U.obscuripennis* and not the holotype of *U.pterosticta* (Fig. 1a); hence, Kashmir should be deleted from locality records of *U.pterosticta*.

It needs to be clarified that the labels of the holotype and paratype (Fig. 1d, e) showed “*Urvaschia pterodiasticta* Hopp” instead of “*Urvaschia pterosticta*” as used in the original description.

### ﻿ Urvaschia obscuripennis

Taxon classificationAnimaliaHemipteraOxycarenidae

(Kiritshenko, 1914) comb. nov.

2011D7D6-A086-5444-893A-430A0FDC89BE

**Figure 3.**
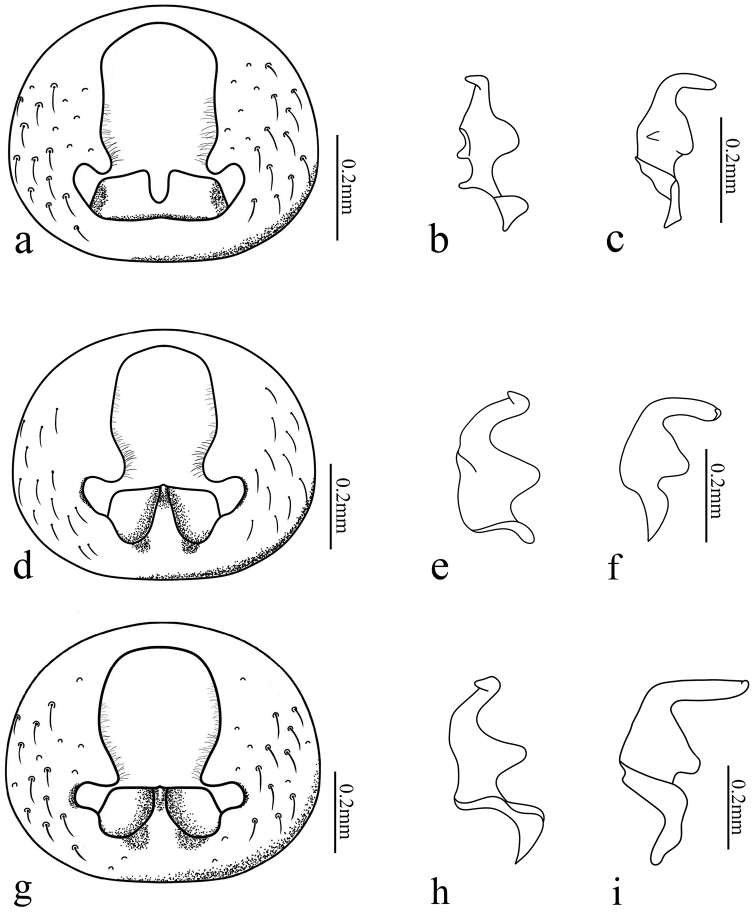
Pygophore (dorsal views, parameres removed) and left paramere (dorsal and lateral views): **a–c***Urvaschiaobscuripennis* comb. nov. **d–f***Urvaschiaconvexa* sp. nov., paratype **g–i***Urvaschiarecta* sp. nov., holotype.

**Figure 4.**
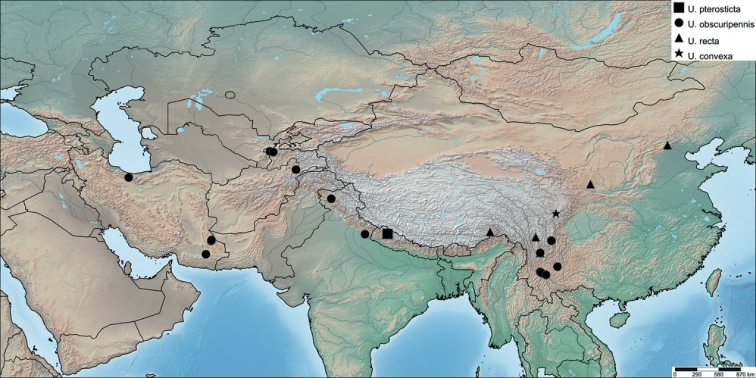
Distribution of the species of *Urvaschia*.

Figs 1b, c, e, f , 2a, d , 3a–c , 4

Camptotelus obscuripennis Kiritshenko, 1914: 411.
Microplax obscuripennis : Muminov 1973: 75; Hoberlandt 1987: 18; Slater and O’Donnell 1995: 76; Péricart 1998: 128; Péricart 1999: 84B: 48; Péricart 2001: 114.

#### Type material examined.

***Lectotype***: Tadzhikistan • ♂; prov. Samarkand. [pr] / Laudan. 17.VII. 12 [hw] / A. N. Kiritshenko. [pr] // Camptotelus / obscuripennis n. sp. // Type [pr, red] // Lectotypus [pr] Camptotelus [hw] / bscuripennis Kir. [hw] / design. [pr] J. Péricart 1993 [hw, red label] (ZIN) (Fig. 1c, f).

***Paratype* of *U.pterosticta* Hopp.** India, Kashmir • ♀; Daksum 9.-13.7. [pr]/ 2400–2700m [pr] // Kashmir, 1976 [pr]/ W. Wittmer [pr] // Urvaschia [hw] / pterodiasticta [hw] / HOPP [hw] / det. Hopp. I. 1987 [hw] // Para- [hw] / Typus [pr] [red label] (NHMB) (Fig. 1b, e).

#### Other material examined.

China• 2♂♂, Yunnan, Yulongshan, Lijiang, Yunnan / 14.vi.1996 / 2700m. leg. Leyi Zheng [all pr] (NKUM); 1♂, Heishui, Yulongshan, Lijiang, Yunnan / 15.vi.1996 / 3000m. leg. Leyi Zheng [all pr] (NKUM); 2♂♂1♀, Shizishan, Wuding, Yunnan / 2200m / 10.viii.1986 [all hw] (NKUM); 2♂♂1♀, Shizishan, Wuding, Yunnan / 2300m / 10.viii.1986 [all hw] (NKUM); 1♀, Yulongshan, Lijiang, Yunnan [all hw] / 13.viii.1979 [hw] / 2800m [hw] leg. Leyi Zheng [pr] (NKUM); 1♀, Yulongshan, Lijiang, Yunnan / 14.viii.1979 / 2700m [all hw] leg. Zuopei Ling [pr] (NKUM); 1♀, Fenghuangshan, Nanjian, Yunnan / 2.xi.2001 / 2400m, leg. Wenjun Bu [all pr] (NKUM); 1♂, Fenghuangshan, Nanjian, Yunnan / 3.xi.2001 / 2400m [all pr] (NKUM); 1♂, Fenghuangshan, Nanjian, Yunnan / 3.xi.2001 / 2400m, leg. Weibing Zhu [all pr] (NKUM); 1♂, Sheyaojing, Wuliang Mountain, Nanjian, Yunnan / 7.xi.2001 / 2400m, leg. Weibing Zhu [all pr] (NKUM); 1♀, Xujiaba, Ailao Mountain [Yunnan, pr] / 82-007466 [hw] / 22.iii.1982 [hw] (NKUM) ; INDIA• 1♂ 1♀ Nainital, / Kumaon, U. P. / India, H. G. C. // Nainital, / W. Almora, / India, H. G. C. // Champion / Coll. B. M. / 1927–409 (BMNH); Afghanistan• 1♂, J. Klapperich / Sarakanda, 3500 m / 26.7.53, Gebirge / Badakschan / NO-Afghanistan [pr] // Microplax ♂ / obscuripennis K [hw] / Det.L.Hoberlandt, 198[pr]4[hw]; 2♀♀, same data except sex [Microplax ♀ / obscuripennis K]; Tadzhikistan 1♂, п б. Искандер- / дарья бл. истоков [=Iskander-darya near source] / *Кириченко* [p] 5 vııı [hw]947 [p] // Microplax / obscuripennis Kir. [hw]; 1♀, р. Сары-таг, оз. / Искандер-куль / *Кириченко* [p] 21 vıı [hw]947 [p]; IRAN• 2♀♀, N. Iran, 4.-9.7 1977 / Kandavan, pass / 3000m, 11.8.70 [p] // Loc. No. 395 / Exped Nat. Mus. / Praha [p] // Microplax ♀ / obscuripennis K [hw] / Det.L.Hoberlandt, 198[p]4[hw]; 1♂, N Iran, C Elburz / Kandavan - pass, / 2700–2900 m, S-slope [p] // Loc. no. 87 / Exp. Nat. Mus. / Praha [p] // Microplax ♂ / obscuripennis K [hw] / Det.L.Hoberlandt, 198[p]4[hw]; 1♀, Энарик – Тамин, / в Кирман, в Перс. / *Зарудн* [p] 21. [hw] VIII98 [p] // Microplax / melanocera n. sp. [hw] / *Oschanin* det. [p].

#### Examined material

**(digital photograph).** China, Sichuan Province, Ganzi Tibetan Autonomous Prefecture, Jiulong County, Wulaxi Town, S215, 28.620355°N, 101.670542°E, photographed by Lu Feng. The image can be found on the iNaturalist website (https://www.inaturalist.org/observations/59187411).

#### Redescription.

***Colouration*.** Head black. Antennae blackish brown, sometimes with segments II and III yellowish brown. Bucculae and labium blackish brown, concolourous with clypeus. Anterior lobe of pronotum black, sometimes with anterior margin yellow; posterior lobe dark blackish brown, with a short yellow midline mark near posterior margin. Scutellum black. Corium pale yellowish brown, with sparse, obscure, pale brown spots except exocorium; veins thick and brown; apical angle of corium with single blackish brown spot. Colour of membrane similar to corium, with dark brown spots between brown veins. Thoracal sterna black. Supracoxal lobe of prosternum blackish brown. Ostiolar peritreme of metathoracic scent gland blackish brown. Posterior 1/2 of mesopleuron and metapleuron broadly yellowish white. Femora blackish brown; tibiae yellow with both ends yellowish brown to blackish brown; colour of tarsi similar to apexes of tibiae. Abdominal sterna dark reddish brown.

***Structure*.** Head slightly declined, both dorsally and ventrally, with very dense, deep, large punctures and erect white setae (ventrally decumbent). Dorsal surface flat. Eyes slightly protruding laterally. Distance between posterior margin of eyes and anterior margin of pronotum ~ 1/2 diameter of eyes. Bucculae high, covering labium, with sparse punctures. Antennae covered with short dense oblique setae; apical one quarter of segment I surpassing clypeus. Labium reaching base of mesocoxae, first segment of labium almost reaching posterior margin of bucculae.

Pronotum trapezoid, flat, calli slightly emergent. Anterior and posterior margin straight; lateral margins of pronotum slightly sinuate; both anterolateral and posterolateral pronotal angles round. Pronotum covered with large, dense punctures (smaller on calli) and long, white, erect setae, slightly leaning posteriad. Base of scutellum sunken, basal 1/2 covered with small punctures and with similar setae as pronotum; apical 1/2 of scutellum without middle ridge, only lateral margins with small punctures. Hemelytra flat, sparsely covered with short white setae; clavus with three clear rows of large shallow punctures, with middle row incomplete. Corium with scattered punctures between vein Cu and clavus, and a row of dense punctures along inner margin of exocorium, apically being superficial, sometimes absent there; apical margin strongly concave, costal margin almost straight; corium evenly broadening posteriad, body broadest near apex of corium; apical angle elongated and narrow; total length of corium ~ 2/3 of hemelytra. Membrane comparatively long, broad, inner ca 1/3 overlapping each other (Figs 1c, 2a); membranal veins thick and obvious; apex of membrane surpassing abdomen. Prosternum, propleura, and lateral part of mesopleura punctate, similarly to pronotum, meso- and metasternum with metapleura impunctate. Ostiolar peritreme of metathoracic scent gland strongly protruding, apically rounded, evaporatorium rounded, reaching > 3/4 over metapleura laterad. Fore femora slightly thickened, sometimes with a tiny spine (Fig. 2d). Abdominal connexivum not exposed. Abdomen impunctate, abdominal sternum covered with sparse setae.

Pygophore: posterior margin of pygophore and cup-like sclerite fused; distal margin of cup-like sclerite with a deep incision (Fig. 3a). Parameres (Fig. 3b, c): outer projection small and rounded; inner projection very small and pointed; blade approximately bent rectangularly to shank of paramere in lateral view.

***Measurements*** (mm, *N* = 8). Body length 2.68–3.50. Head length 0.34–0.47, width across eyes 0.60–0.72; antennal segments I–IV length: 0.17–0.26: 0.37–0.56: 0.27–0.32: 0.40–0.45; labium length 0.96, first segment length 0.23. Pronotum length 0.55–0.68, width of anterior margin 0.48–0.54, width of posterior margin 0.68–0.88; scutellum length 0.31–0.39, width 0.33–0.50. Distance apex clavus-corium apex 0.74–0.97; distance apex corium – apex membrane 0.70–0.88.

#### Distribution.

China (Sichuan, Yunnan); India (Kashmir, Uttarakhand) (Hopp 1987); Iran (Alborz, Sistan and Baluchestan); Afghanistan (Badakshan); Tadzhikistan (Fig. 4).

#### Remarks.

As mentioned above, the female paratype of *U.pterosticta* from Kashmir (Fig. 1b) was transferred to this species. Meanwhile, the original distribution information from China of this species should be considered a misidentification; see the detailed comments of *Urvaschiaconvexa* sp. nov.

#### Differential diagnosis.

*Urvaschiaobscuripennis* is similar to *U.pterosticta* in having similar brown spots on the hemelytra and apex of the corium conspicuously concave, but the lateral margin of the corium is almost straight and the exocorium spotless, the lateral margins of pronotum are slightly sinuate (vs. lateral margin of the corium more arched and exocorium with brown spots; the lateral margins of the pronotum more arched in *U.pterosticta*), and the distal margin of the cup-like sclerite with a deep incision (vs. distal margin of cup-like sclerite without any incision but with a median keel in *U.pterosticta*).

### ﻿ Urvaschia convexa sp. nov.

Taxon classificationAnimaliaHemipteraOxycarenidae

FF7CDB60-C5FB-54C8-AB10-551A62E68C34

https://zoobank.org/4060CA49-013C-4BE2-80C6-2353010E1247

Figs 2b, e , 3d–f , 4

Camptotelus obscuripennis : Zheng and Zou 1981: 91, fig. 323, pl.13: 129. Misidentification.

#### Type material examined.

***Holotype*.** China • ♀; Maerkang [pr], Sichuan [pr] / 2600–2800m / 13.viii.1963 [hw] (TNHM) // leg. Jiang Xiong [pr] // *Camptotelus obscuripennis* Kiritshenko [hw]/ det. Leyi Zheng [hw]. ***Paratype*.** China • ♂; Maerkang [pr], Sichuan [pr] / 2600–2800m / 11.viii.1963 [hw] (TNHM) // leg. Jiang Xiong [pr].

#### Description.

***Colouration*.** Head blackish brown; bucculae and labium brown; antennae dark brown. Pronotum brown, with a pale brown midline; callar area of pronotum blackish brown. Scutellum blackish brown, distal 3/4 with a brown midline. Corium pale yellowish brown, evenly covered with obscure brown spots; distal margin of corium dark brown; apical angle of corium with a small blackish brown spot. Colour of membrane similar to that of corium, with brown spots between brown veins. Thoracic sterna blackish brown except posterior 1/2 of prosterna yellowish brown. Supracoxal lobe of prosternum yellow. Mesopleuron blackish brown; inner 1/2 of ostiolar peritreme of metathoracic scent gland yellowish white, outer 1/2 of ostiolar peritreme brown. Posterior 1/2 of metapleura broadly yellowish white. Abdominal sterna dark reddish brown. Femora dark brown, tibiae ochraceous.

***Structure*.** Head slightly declined, covered very densely with deep and large punctures and long white erect setae both dorsally and ventrally; vertex comparatively flat. Eyes slightly protruding laterally. Distance between posterior margin of eyes and anterior margin of pronotum ~ 1/3 of diameter of eyes. Bucculae high, covering labium, with sparse punctures. Antennae covered with dense oblique setae; apical quarter of segment I surpassing clypeus. Labium reaching base of mesocoxae, first segment of labium surpassing posterior margin of bucculae. Venter of head comparatively flat, covered with dense punctures and dense decumbent setae.

Pronotum trapezoid, flat, calli slightly emergent. Anterior margin straight; middle part of posterior margin slightly concave; lateral margins of pronotum sinuate; both of anterolateral and posterolateral pronotal angles rounded. Pronotum covered with dense punctures and with long erect setae, slightly leaning posteriad. Base of scutellum slightly sunken, covered with punctures and setae except midline, slightly emergent in apical 1/2. Hemelytra flat, sparsely covered with white short setae; clavus with inner and outer rows of strong and shallow punctures, scattered with many irregular punctures between them. Corium with sparse scattered punctures. Apical margin of corium convex, costal margin evenly arched; body broadest near apex of clavus, length of corium almost three fourth of hemelytra. Membrane short and small, only overlapping each other on inner edge (Fig. 2b); membranal veins remarkable; apex of membrane surpassing tip of abdomen. Ostiolar peritreme of metathoracic scent gland strongly protruding, apically rounded. Fore femora slightly thickened, unarmed (Fig. 2e). Abdominal connexivum not exposed. Abdominal sternum impunctate, covered with comparatively dense setae.

Pygophore (Fig. 3d): Posterior margin of pygophore and cup-like sclerite fused. Parameres (Fig. 3e, f): outer projection large and slightly sharp; inner projection very small; blade nearly rectangularly bent to shank of paramere in lateral view.

***Measurements*** (mm, *N* = 2). ***Holotype*.** ♀ (***Paratype*.** ♂), Body length 3.44 (3.08). Head length 0.53 (0.45), width across eyes 0.65 (0.69); antennal segments I–IV length: 0.20: 0.50: 0.36: 0.41 (0.17: 0.42: 0.31: 0.43); labium length 1.26 (covered), first segment length 0.31. Pronotum length 0.71 (0.66), width of anterior margin 0.58 (0.53), width of posterior margin 1.01 (0.85); scutellum length 0.44 (0.39), width 0.57 (0.40). Distance apex clavus– apex corium 1.03 (0.92); distance apex corium–apex membrane 0.76 (0.65).

#### Etymology.

The species epithet, *convexa*, is an adjective and refers to the convex distal margin of corium.

#### Distribution.

China (Sichuan) (Fig. 4).

#### Differential diagnosis.

Based on the description and figures, we conclude that the new species was always misidentified as “*Camptotelusobscuripennis* Kiritshenko, 1914” in China (Zheng and Zou 1981, 1987; Zheng 1988). When we examined the photographs of the type of *Camptotelusobscuripennis*, we found they are different but closely related species. The new species differs from *U.obscuripennis* in the following combination of characters: antennae unicolourous (vs. antennae not unicolorous in *U.obscuripennis*); distal margin of corium convex and apical angle of corium not elongated (vs. distal margin of corium concave; apical angle elongated and pointed in *U.obscuripennis*); inner 1/2 of ostiolar peritreme of metathoracic scent gland yellowish white, outer 1/2 brown (vs. ostiolar peritreme of metathoracic scent gland black in *U.obscuripennis*); profemur unarmed (vs. profemur with a spine).

### ﻿ Urvaschia recta sp. nov.

Taxon classificationAnimaliaHemipteraOxycarenidae

CC807A6F-D1A8-572D-A515-6F13768E517C

https://zoobank.org/E6FF0F07-0BAB-4014-B183-858C6A2FE4F8

Figs 2c, f , 3g–i , 4

#### Type material examined.

***Holotype*.** China • ♂; Lijiang [hw], Yunnan [pr] / 11.viii. [hw]1979 [pr] / leg. Jianxin Cui [pr] (NKUM). ***Paratypes*.** China • 1♀, Bayi town, Xizang / 6.viii.2003 / leg. Huaijun Xue, Xinpu Wang [all pr] (NKUM); 1♀, Xiaonanchuan Forestry Centre, Erlonghe, Liupanshan, Ningxia / 28.vi.2008 / 1900m. leg. Gengping Zhu [all pr] (NKUM); 1♀, Zhongreniao, Xiangcheng [all hw], Sichuan [pr] / 3950m // 1982.VII.4 [all hw] / leg. Huaicheng Chai [hw] (IZAS).

#### Other material examined.

China • pr. Beijing / Mentougou Dist. / Beijing 130 km NW / Liyan Ling // Linshan Mt. / 1749 m, 115°30'E / 40°00', 2.VIII.2002 // leg. G. Melika (HNHM); NEPAL• 1♂ 1♀ Umg. Alm Darghari / b. Maharigaon, 4000m // Gebiet von Jumla / Westnepal, lg. H. Franz // COLLECTION / ERNST HEISS / Innsbruck – Austria (CEHI in TLMF).

#### Description.

***Colouration*.** Head blackish brown. Antennae dark blackish brown. Bucculae and labium dark brown. Pronotum with a yellowish white midline except area of calli. Anterior lobe of pronotum blackish brown, posterior lobe dark brown. Scutellum blackish brown. Hemelytra pale yellowish brown, with dense dark brown spots between brown veins covering exocorium as well; distal margin of corium dark brown; apical angle of corium with a blackish brown spot. Thoracal sterna blackish brown. Supracoxal lobes yellowish white to yellow. Mesopleuron black; inner 1/2 of ostiolar peritreme of metathoracic scent gland yellowish white, outer 1/2 of ostiolar peritreme brown. Posterior 1/2 of metapleura broadly yellowish white. Femora blackish brown; tibiae and tarsi ochraceous. Abdominal sterna dark reddish brown.

***Structure*.** Head slightly declined, covered with large deep punctures and erect white setae. Eyes slightly protruding laterally. Distance between posterior margin of eyes and anterior margin of pronotum 1/2 diameter of eyes. Bucculae high, almost parallel to labium, visible laterad of clypeus from dorsal view. Antennae covered with dense oblique setae, apical 1/3 of segment I surpassing clypeus. First segment of labium surpassing bucculae, segment II surpassing base of head, labium reaching middle of mesocoxae. Venter of head flat, covered with punctures and dense white decumbent setae.

Pronotum trapezoid, flat, covered with large dense punctures and long white erect setae, slightly leaning posteriad; calli slightly emergent. Anterior margin of pronotum straight; posterior margin of pronotum straight with posterolateral pronotal angles slightly protruding posteriad. Base of scutellum slightly sunken; each margin covered with dense punctures, smaller than on pronotum and sparse setae, central area with sparse punctures and inconspicuous median carina. Hemelytra flat, sparsely covered with white and short setae; clavus with inner and outer rows of punctures, scattered with irregular one or two rows of punctures in middle. Corium with several punctures between vein Cu and clavus, and a row of punctures along inner margin of exocorium (Fig. 2c); cubital vein inconspicuous. Apical margin of corium straight, costal margin evenly arched; body broadest near apex of clavus; corium longer than 2/3 of hemelytra. Membrane comparatively broad, almost fully overlapping each other (Fig. 2c); membranal veins thick and conspicuous; apex of membrane surpassing abdomen. Femora slightly thickened, profemora sometimes with one small spine (Fig. 2f). Abdominal connexivum not exposed. Abdominal sternum impunctate, covered with sparse setae.

Pygophore (Fig. 3g): Posterior margin of pygophore and cup-like sclerite fused. Parameres (Fig. 3h, i): outer projection large, triangular; inner projection inconspicuous; blade bent rectangularly with shank of paramere from lateral view.

***Measurements*** (mm, *N* = 3). ***Holotype*.** ♂ (***Paratypes***. 2♀♀); Body length 3.49 (3.72–3.81). Head length 0.46 (0.50–0.51), width across eyes 0.77 (0.73–0.76); antennal segments I–IV length: 0.22: 0.46: 0.33: 0.40 (I–IV: 0.18–0.25: 0.54: 0.35: 0.45); labium length 1.34, first segment length 0.32. Pronotum length 0.73 (0.73–0.76), width of anterior margin 0.60 (0.64), width of posterior margin 1.04 (1.12–1.15); scutellum length 0.51 (0.48–0.50), width 0.56 (0.67–0.71). Distance apex clavus– apex corium 1.08 (1.08–1.18); distance apex corium–apex membrane 0.78 (0.98–1.05).

#### Etymology.

The species epithet *recta*, derived from Latin adjective *rectus* (= straight), alludes to the straight apical margin of the corium.

#### Distribution.

China (Beijing, Ningxia, Sichuan, Xizang, Yunnan), Nepal (Fig. 4). The locality of the Nepalese specimen is very near to the type locality of *U.pterosticta*; therefore, it cannot be seen separately on Fig. 4.

#### Differential diagnosis.

The new species is similar to *U.convexa* sp. nov. in having brown spots on the hemelytra and oval body shape, but the corium is not elongated, with the length of the corium almost twice the length of the membrane from the apical angle of the corium to the apex, and its apex is almost straight (vs. corium conspicuously elongated, with the length of the corium almost three times the length of the membrane from the apical angle of the corium to the apex, and the apex of the corium is conspicuously convex in *U.convexa* sp. nov.); membrane almost fully overlapping (vs. membrane only overlapping on the inner edge in *U.convexa* sp. nov.).

### ﻿Key to species of *Urvaschia*

**Table:** 

1	Distal margin of corium markedly concave, apex of corium elongated (Figs 1, 2a)	**2**
–	Distal margin of corium convex or straight, apex of corium not elongated (Fig. 2b, c)	**3**
2	Costal margin of corium convex, with brown spots; lateral margin of pronotum slightly arched, not sinuate (Fig. 1a); distal margin of cup-like plate carinate, acute	***U.pterosticta* Hopp, 1987**
–	Costal margin of corium straight, excorium unicolourous, spotless; lateral margin of pronotum slightly sinuate (Fig. 2a); distal margin of cup-like plate with a deep incision (Fig. 3a)	***U.obscuripennis* (Kiritshenko, 1914) comb. nov.**
3	Distal margin of corium convex; membrane only overlapping on inner edge (Fig. 2b)	***U.convexa* sp. nov.**
–	Distal margin of corium almost straight; membrane fully overlapping each other (Fig. 2c)	***U.recta* sp. nov.**

## ﻿Discussion

Until now, the regional Palaearctic *Urvaschia* species seemed to be endemic in Nepal and Kashmir, but four species distributed in six countries documented here indicate a more widely-distributed taxon. It is interesting that the shape of the corial apical margin of *Urvaschia* species varies between different species, from concave, straight, to convex. This demands further investigations using both morphological and molecular evidence of species of related oxycarenid genera.

## Supplementary Material

XML Treatment for Urvaschia

XML Treatment for Urvaschia pterosticta

XML Treatment for Urvaschia obscuripennis

XML Treatment for Urvaschia convexa

XML Treatment for Urvaschia recta
